# High‐Entropy Chemical Composition Design for Ultrahigh Capacitive Energy Storage

**DOI:** 10.1002/advs.202521163

**Published:** 2025-12-03

**Authors:** Muhammad Habib, Haoyu Wang, Weisan Fang, Attaur Rahman, Maqbool Ur Rehman, Ting Wang, Qingfeng Zhu, Muhammad Javid Iqbal, Xiaoming Shi, He Qi, Weiping Gong

**Affiliations:** ^1^ Guangdong Provincial Key Laboratory of Electronic Functional Materials and Devices Huizhou University Huizhou Guangdong 516001 China; ^2^ School of Mathematics and Physics University of Science and Technology Beijing Beijing 100083 China; ^3^ School of Materials Science and Engineering Anhui Polytechnic University Wuhu 241000 China; ^4^ Department of Physics University of Peshawar Peshawar 25120 Pakistan; ^5^ School of Materials Science and Engineering Hainan University Haikou Hainan 57228 China

**Keywords:** high‐entropy ceramics, energy storage, polar nanoregions, polymorphic domains

## Abstract

Superior energy‐storage performance is imperative for next‐generation electronics and pulsed power systems. However, in lead‐free dielectric ceramics, achieving synergistic optimization of energy storage performance remains a critical challenge. The central obstacle lies in the simultaneous enhancement of both energy density (*W*_rec_) and efficiency (*η*). Herein, a high‐entropy lead‐free Bi_0.32_Na_0.32_Ba_0.32_La_0.04_TiO_3_‐NaNbO_3_ ceramics system is designed, guided by phase‐field simulations and a strategic chemical composition approach. The synergistic effect of La‐donor doping and NaNbO_3_ addition induces local random fields and local random stresses, stabilizing multiphase ultrasmall polar nanoregions. Benefiting from these features, an ultrahigh energy storage performance of *W*_rec_ ≈ 12.4 J cm^−^^3^ and *η* ≈ 82.6% is realized under the applied field 790 kV cm^−1^. Furthermore, a large *W*_rec_ of ≈6.2 J cm^−3^ and a high *η* ≈ 85.8% with less than 5% variation across a broad temperature (30–150 °C) range are highly promising results for lead‐free ceramics. This study marks a significant advancement in the energy storage performance and also provides a paradigm for the development of new lead‐free ceramics for the next generation of pulsed power applications.

## Graphical abstract

La‐donor doping suppresses defect charges and reduces domain size via local random fields. Conversely, NaNbO_3_ addition in BNT‐based ceramics stabilizes the orthorhombic phase. This creates R‐O‐T multiphase polar nanoregions within a cubic matrix, which lowers the energy barrier, delays polarization saturation, and enables a high breakdown strength, leading to superior energy storage performance.

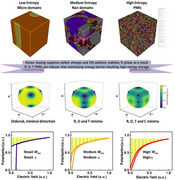

## 1 Introduction

Dielectric capacitors are indispensable for applications requiring ultra‐high power density and ultrafast charge–discharge rates, such as pulsed power systems, medical devices, data storage devices, microwave telecommunication, and electric vehicles.^[^ ^1^, ^2^, ^3^, ^4^, ^5^ ^]^ The recoverable energy density (*W*_rec_) and efficiency (*η*) are primarily determined from the discharge area of the polarization‐electric field (*P*–*E*) hysteresis loop and are fundamentally governed by the material's maximum polarization (*P*_m_), remnant polarization (*P*_r_), and breakdown field (*E*_B_).^[^ ^6^ ^]^ Recently, exceptional ultrahigh *W*_rec_ = 14.8 J cm^−3^ and *η* ≈ 90.2% were achieved at 860 kV cm^−1^ in 6% of La‐doped Pb(Zr_1/3_Sn_1/3_Hf_1/3_)O_3_ relaxor antiferroelectrics ceramics due to medium entropy configuration.^[^ ^1^ ^]^ Despite their superior performance, the toxicity of lead poses threats to human health and the environment, leading to severe global regulations on the use of lead‐based materials. Consequently, it will be a great progress to enhance the energy storage performance of the lead‐free materials to integrate the dielectric capacitor for advanced pulse power technology.

The high‐entropy relaxor ferroelectrics are the core material for capacitor energy storage properties.^[^ ^2^, ^4^, ^5^, ^6^, ^7^, ^8^, ^9^, ^10^, ^11^, ^12^, ^13^ ^]^ In general, the lead‐free, BaTiO_3_ (BT), BiFeO_3_ (BF), (Bi_0.5_K_0.5_)TiO_3_ (BKT), and (Bi_0.5_Na_0.5_)TiO_3_ (BNT) ceramics are strong candidates due to their large *P*_m_ > 50 μ cm^−2^. Nonetheless, its energy storage application is severely limited by its strong ferroelectric nature, which manifests in a large *P*_r_ and high coercive field (*E*_c_). It is well established that hysteresis loss indicates the energy dissipation, which raises the internal heat of the sample and limits their *E*_B_ < 200 kV cm^−1^.^[^ ^3^, ^14^ ^]^ A well‐established strategy is chemical modification to further disrupt the long‐range ferroelectric order and induce local structure heterogeneity that stabilizes the short‐range relaxor ferroelectric order.^[^ ^2^, ^4^, ^15^, ^16^, ^17^, ^18^, ^19^ ^]^ The high‐entropy relaxor ferroelectric compositions design is an emerging idea for the improvement of energy storage performance.^[^ ^9^, ^12^, ^14^, ^20^ ^]^ Until now, some interesting results (*W*_rec_ ≥ 10 J/cm and *η* ≥ 80%) have been achieved in the high‐entropy lead‐free ceramics.^[^ ^2^, ^3^, ^4^, ^5^, ^6^, ^9^, ^12^, ^14^, ^20^, ^21^, ^22^, ^23^, ^24^, ^25^, ^26^, ^27^, ^28^, ^29^, ^30^, ^31^ ^]^ It is commonly accepted that the relaxor ferroelectric characteristics in perovskite material originate from multi‐element modifications.^[^ ^27^ ^]^ The co‐occupation of different atoms on the same crystallographic site induces random stress/field due to their unlike ferroelectric activity, valence states, and ionic radii, which strongly affect the local symmetry.^[^ ^32^ ^]^ However, multi‐element complex modifications have the risk of poor reproducibility for mass production in industry.^[^ ^33^ ^]^ Thus, it will be a great progress for the industry and also rewarding scientifically to design a simple composition by using a feasible approach and achieve *W*_rec_ ≥ 10 J cm^−1^ in lead‐free relaxor ferroelectric ceramics, which is comparable to the lead‐based ceramics. Hence, the strategic approach of chemical composition design is very important for high‐energy storage properties.^[^ ^23^, ^25^, ^26^ ^]^

The (1‐*x*)BNT‐*x*BT solid solutions have been extensively studied, and a concentration of ≈6% BT develops a classical morphotropic phase boundary (MPB) between rhombohedral (R) and tetragonal (T) phases, which is renowned for enhancing piezoelectric response.^[^ ^34^ ^]^ However, the compositions beyond the MPB with high concentrations of BT (≈30%) induce pronounced relaxor characteristics, leading to a significant reduction in *P*_r_ and a slimmer *P*–*E* loop, which is highly beneficial for energy storage.^[^ ^27^ ^]^ Mostly in relaxor ferroelectrics, the critically low practical breakdown strength is associated with its defect chemistry that induces high electrical conductivity. To further optimize this system, a donor doping strategy has been employed. The A‐site substitution, Ba^2^⁺ or (Bi,Na)^2⁺^ by La^3^⁺ introduces compositional heterogeneity through two key mechanisms: i) a local random field due to charge imbalance, and ii) local random stress due to ionic radius mismatch. This combined effect effectively suppresses the formation of macro‐domains, stabilizes polar nano‐regions (PNRs), and increases configurational entropy.^[^ ^4^, ^5^, ^35^ ^]^ This high‐entropy state promotes a diffuse phase transition and a highly slimmer hysteresis loop, which is ideal for achieving high *W*_rec_ and *η*. Furthermore, donor doping can also suppress oxygen vacancies and associated leakage currents, thereby significantly improving the breakdown strength (BDS), which is a crucial factor for enhancing energy density.^[^ ^5^ ^]^ Consequently, the introduction of antiferroelectric (AFE) characteristics is another powerful route to improve energy storage, as AFEs exhibit near‐zero *P*_r_ and a large energy difference between charge and discharge cycles.^[^ ^1^, ^36^, ^37^ ^]^ Previously, it was found that the incorporation of NaNbO_3_ (NN) orthorhombic (O) phase into the BNT or BKT matrix yields AFE‐like characteristics that significantly decrease *P*_r,_ which is beneficial for energy storage.^[^ ^27^, ^38^ ^]^ This synergic approach of relaxor state fostered by La‐donor doping and the AFE characteristics prompted by NN addition is anticipated to simultaneously maximize *W*_rec_ and *η* of the BNT‐based ceramics.

## 2 Results and Discussion

**Figure 1:**
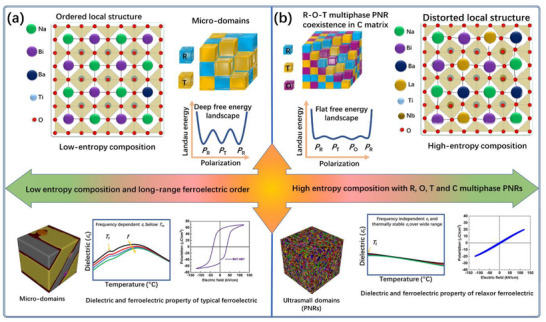
Schematic diagram of the strategic composition design for excellent energy storage performance. a) Low‐entropy composition atomic arrangement with micro‐domains and a deep free energy landscape with R and T phase boundary, typical ferroelectric dielectric and polarization response. b) High‐entropy composition, high local structure heterogeneity, and a flat free energy landscape due to multiphase PNRs, relaxor ferroelectric *P*–*E* loop, and temperature‐dependent dielectric constant.

In this work, we design and fabricate a novel lead‐free ceramic system according to the chemical formula (1‐*x*) Bi_0.32_Na_0.32_Ba_0.32_La_0.04_TiO_3_‐*x*NaNbO_3_ (BNBLT‐*x*NN, with *x* = 0.0, 0.1, 0.2, 0.3, and 0.4). The La‐donor doping induces heterogeneity and also suppresses charge carrier mobility, while NN addition stabilizes the O phase. This synergistic approach causes domain miniaturization and stabilizes R‐O‐T polymorphic PNRs in the C matrix. These ultrasmall randomly oriented PNRs delay polarization, which substantially contributes to the breakdown strength. **Figure** **1** illustrates the strategic approach for designing high‐entropy compositions to optimize energy storage performance. The compositional inhomogeneity can be quantified from the entropy configuration, which is defined as Δ*S*_conf_ = ‐*R*[$\sum_{i=1}^{N}x_{i}lnx_{i}$+$\sum_{j=1}^{M}x_{j}lnx_{j}$], where *R* is the ideal gas constant while *N*, *M*, *x*_i_, and *x*_j_ represent the A‐site and B‐site atomic species and contents, respectively.^[^ ^28^ ^]^ For a comparative study, a low entropy (Δ*S*_conf_ = 0.875*R*) composition is designed near the MPB according to the chemical formula 0.94(Bi_0.5_Na_0.5_)TiO_3_‐0.06BaTiO_3_ (BNT‐6BT) as given in Table S1 (Supporting Information) in the supporting information. As shown in Figure S1a (Supporting Information), the entropy configuration of the BNBLT‐*x*NN system gradually increased with NN addition, reached Δ*S*_conf_ = 1.72*R* at *x* = 0.3, and then decreased at higher *x* ≥ 0.4 values. The NN is added into BNBLT, where the Na⁺ concentration on the A‐site increases disproportionately. At *x* = 0.3, the A‐site cation ratio becomes approximately Na:Bi:Ba:La ∷ 52:22:22:4, where the Na⁺ fraction surpasses 50%, the system begins to deviate from an ideal, maximally random mixture of multiple cations. With further increase in NN content (*x* > 0.3), the A‐site becomes increasingly dominated by a single cation (Na⁺), which reduces the configurational entropy. According to the phenomenological relation, the highest energy storage can be expected for this high entropy Δ*S*_conf_ = 1.72*R* at *x* = 0.3 composition. More detailed calculation for the entropy configuration is given in Tables S1–S7 (Supporting Information). Figure 1a represents a low entropy composition that exhibited micro‐domains with a relatively deep energy profile. A strong frequency‐dependent dispersion in the dielectric permittivity below the Curie temperature (*T*_C_) and a typical ferroelectric *P*–*E* curve are the characteristics of low entropy composition. The incorporation of La donor and NN into a BNT‐based ceramics induces local structure heterogeneity, due to occupation of distinct atoms on the A‐site (Na^1+^, Bi^3+^, Ba^2+^, and La^3+^) and B‐site (Ti^4+^, Nb^5+^) [Figure 1b]. This highly distorted local structure produces abundant ultrasmall‐sized nano‐domains due to critically high Δ*S*_conf_ ≈1.72*R* as predicted during composition design. These randomly oriented multiphase PNRs reduce polarization anisotropy and delayed polarization saturation, leveraging energy storage performance. We systematically investigate the effects of this dual strategy on the phase structure, domain morphology, dielectric relaxation, and ferroelectric properties for energy storage applications. The design composition shows a high potential for next‐generation, environmentally friendly high‐pulse power capacitor applications.

**Figure 2:**
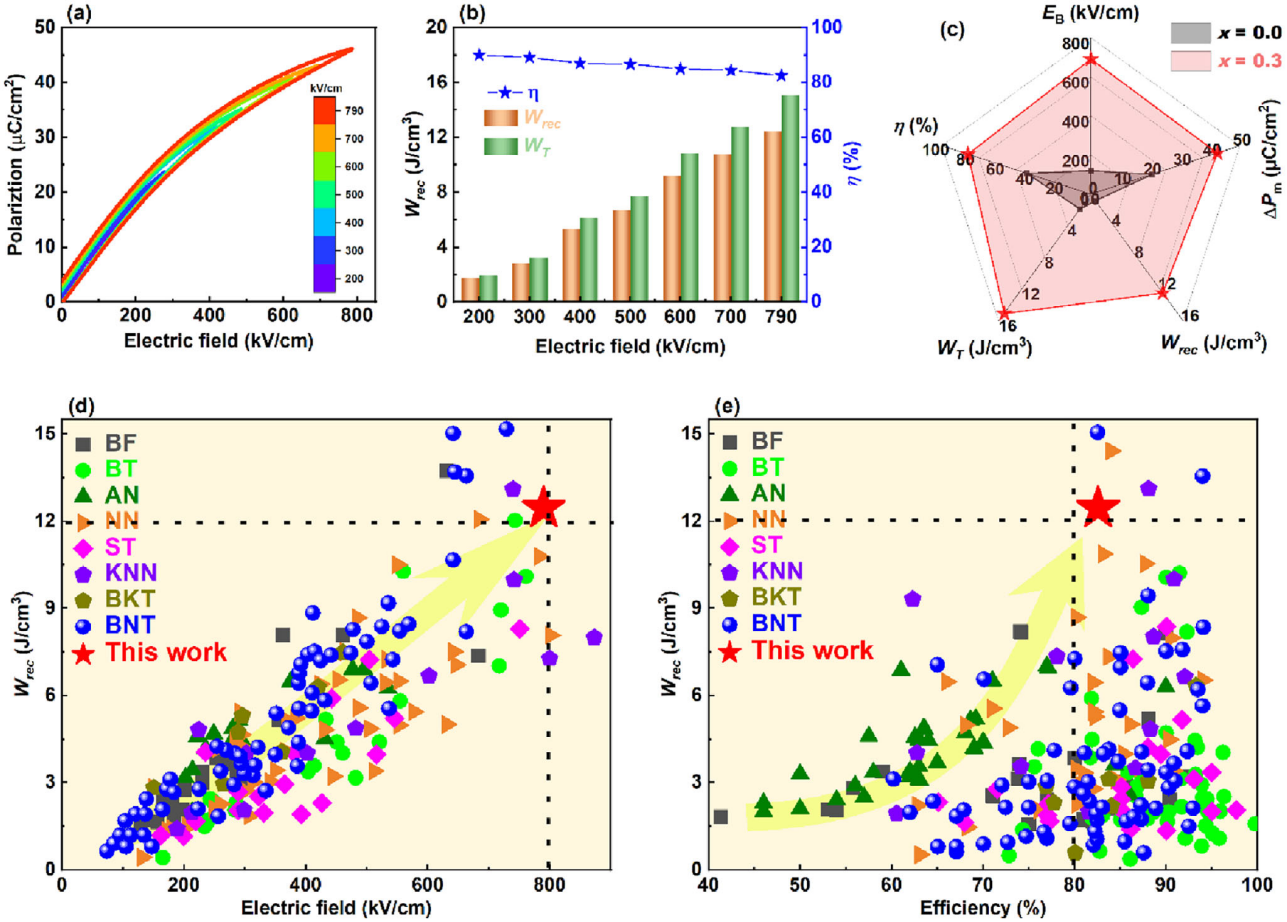
High‐entropy composition design and energy storage performance. a) Field‐dependent unipolar *P*–*E* loops and b) characteristic parameters of energy storage property (*W*_rec_, *W*_T_, and *η*) for *x* = 0.3 ceramic. c) Radar chart comparing key performance metrics (*E*_B_, Δ*P, W*_rec_, *W*_T_, and *η*) of *x* = 0.0 and *x* = 0.3 samples. d,e) Comparison of *W*_rec_, *E*_B_, and *η* with other reported lead‐free bulk ceramics including BiFeO_3_ (BF), BaTiO_3_ (BT), AgNbO_3_ (AN), NaNbO_3_ (NN), SrTiO_3_ (ST), (K, Na)NbO_3_ (KNN), (Bi, K)TiO_3_ (BKT), and (Bi, Na)TiO_3_ (BNT).^[^ ^2^, ^5^, ^9^, ^12^, ^20^, ^21^, ^22^, ^23^, ^24^, ^25^, ^26^, ^27^, ^28^, ^29^, ^30^, ^31^ ^]^

Ultrahigh energy storage performance is demanded for cutting‐edge advanced pulse power technology. High entropy is an emerging idea for the improvement of energy storage properties.^[^ ^4^, ^6^, ^12^ ^]^ According to phenomenological relation and previous investigations, the entropy configuration is classified into low‐entropy (Δ*S*_conf_ < 1.0 *R*), medium‐entropy (1.0*R* ≤ Δ*S*_conf_ ≤ 1.5*R*), and high‐entropy (Δ*S*_conf_ > 1.5*R*).^[^ ^10^ ^]^ High entropy in bulk ceramics produces polymorphic PNRs, which are randomly distributed and cause delayed polarization saturation, improving energy storage performance.^[^ ^2^ ^]^ Figure S1b (Supporting Information) represents the bipolar *P*–*E* loops for BNT‐6BT and BNBLT‐*x*NN with *x* = 0.0, 0.1, 0.2, and 0.3 under 120 kV cm^−1^. A strong polarization response (*P*_r_ > 50 µC cm^−2^ and *P*_m_ ≈ 68 µC cm^−2^) of the BNT‐6BT is mainly related to microdomains. In the case of the BNBLT‐*x*NN system, the *x* = 0.0 and *x* = 0.1 ceramics illustrate early polarization saturation due to lower Δ*S*_conf_ < 1.5*R* from the critical value. On the other hand, the *x* ≥ 0.2 compositions are situated in the high‐entropy region, which displayed super‐paraelectric relaxor *P*–*E* loops. Based on the composition design, *x* = 0.3 is a relaxor ferroelectric with the highest entropy, which is the most suitable candidate for energy storage properties. The random distribution of the multiple‐phase PNRs in the high‐entropy compositions delays polarization saturation and ultimately improves energy storage.^[^ ^26^ ^]^ Therefore, field‐dependent unipolar *P*–*E* loops of the optimal composition (*x* = 0.3) were measured up to their *E*_B_ ≈ 790 kV cm^−1^ as given in **Figure** **2**a. This high breakdown strength can be attributed to the ultrafine grain structure with high grain boundary density and the presence of randomly oriented multiphase ultrasmall PNRs.^[^ ^26^ ^]^ Consequently, the improved breakdown strength resulted in an ultrahigh energy storage performance for the *x* = 0.3 sample, with *W*_rec_ = 12.4 J cm^−3^, *W*_T_ = 15.6 J cm^−3^ and *η* ≈ 82.6% [Figure 2b]. Encouragingly, the *W*_rec_ and *η* are higher than most reported lead‐free materials and comparable to the state‐of‐the‐art energy storage dielectric ceramics.^[^ ^6^, ^12^, ^22^, ^28^, ^31^ ^]^ Figure 2c provides a holistic comparison in a radar chart of the key parameters (*E*_B_, Δ*P*, *W*_rec_, *W*_T_, and *η*) for *x* = 0.0 and 0.3 ceramics. Interestingly, the high‐entropy ceramic (*x* = 0.3) exhibited comprehensive superiority in energy storage performance over the medium‐entropy composition. Our material achieves an overall energy storage performance, *W*_rec_ ≈ 12.4 J cm^−3^ and *η* ≈ 82% with *E*_B_ ≈ 790 kV cm^−1^ that surpasses most recently reported lead‐free systems.^[^ ^12^, ^14^, ^18^, ^22^, ^28^, ^39^ ^]^ As shown in Figure 2d,e, only a few systems are located at a performance boundary.^[^ ^2^, ^5^, ^9^, ^12^, ^20^, ^21^, ^22^, ^23^, ^24^, ^25^, ^26^, ^27^, ^28^, ^29^, ^30^, ^31^ ^]^ Our work demonstrates significant progress in dielectric energy storage, and the high‐entropy composition design provides a paradigm for future research. To correlate the energy storage performance of this work with the phase structure, the XRD patterns were examined [Figure S2a,b, Supporting Information]. In the (200) peak, the splitting indicates T dominant structure. This peak gradually disappears for the higher order of NN content (*x* > 0.1), confirming the emergence of the pseudo‐cubic phase.

**Figure 3:**
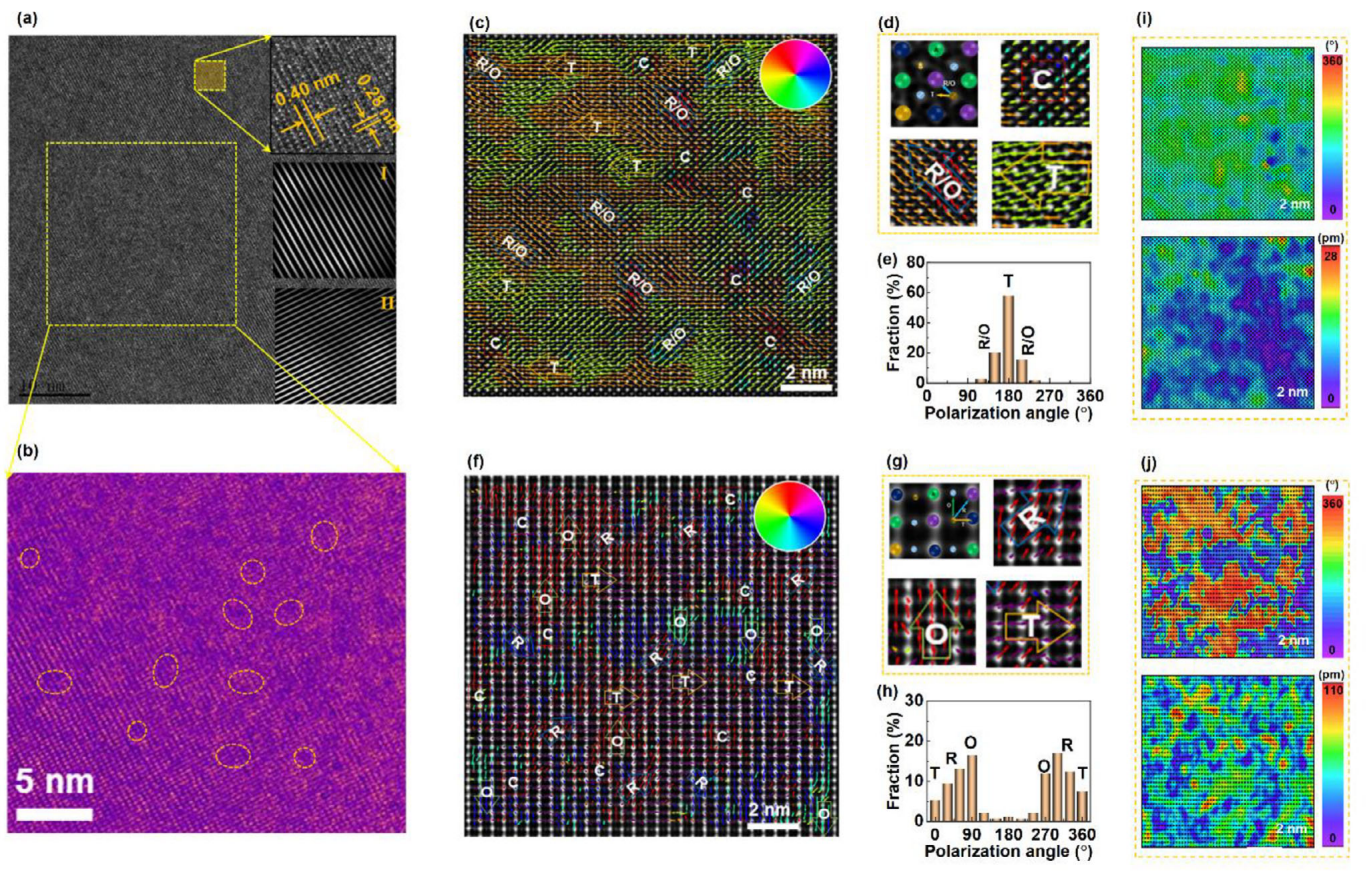
Atomic‐scale local structure for BNBLT‐*x*NN with *x* = 0.3 ceramics. a,b) TEM image of domain morphology. c) Atomic resolution HAADF‐STEM images captured along the [100] axis with polarization histogram and d) magnified view for the PNRs of C, R/O, and T symmetries. e) Statistical distribution of polarization vectors or R/O and T phases calculated from [100] image. f) The HAADF‐STEM image was taken along the [110] axis, and g) the enlarged view of R, O, and T local structure. h) Statistical distribution of polarization vectors or R, O, and T phases calculated from [110] image. Atomic polarization angle mapping and polarization magnitude mapping for i) [100] and j) [110] images calculated from (c) and (f), respectively.

The achievement of excellent energy storage properties is closely related to the domain configuration.^[^ ^28^, ^40^ ^]^ To explore the domain structure evolution and origin of high energy storage, the PFM measurements are conducted for *x* = 0.0 and 0.3 ceramics. The PFM images in Figure S3 (a_1_) (Supporting Information) show that the *x* = 0.0 composition consists of relatively large‐sized domains. Although the micro/nano‐domains displayed a large *P*_m_ (a critical parameter for excellent energy storage) but the concurrent high *P*_r_ value resulted in poor efficiency. Generally, the large hysteresis of the low/medium‐entropy compositions is mainly ascribed to irreversible switching of large‐sized ferroelectric domains.^[^ ^28^ ^]^ The NN addition into BNBLT ceramics increases the compositional disorder degree, and at *x* = 0.3, the Δ*S*_conf_ is raised above the critical value. This high entropy composition comprises ultrasmall‐sized domains, which are beyond the resolution limit of the PFM, as shown in Figure S3 (b_1_) (Supporting Information). Such ultrasmall‐sized domains, known as PNRs, have been previously observed in high entropy ceramics.^[^ ^38^, ^39^, ^40^ ^]^ The PNRs respond more quickly to the external stimulus, leading to high capacitive energy storage performance. Previous investigations have confirmed that micro/nano‐domains show partial switching while PNRs are uniformly switched under the DC‐bias field.^[^ ^41^, ^42^ ^]^ To further investigate the presence of PNRs in the high entropy composition, we performed PFM measurements under a DC‐bias field. Figure S3 (a_2_) (Supporting Information) shows the PFM amplitude and phase images taken under ±20 V for *x* = 0.0 sample. A diverse color distribution indicates that the applied voltage is insufficient for complete domain switching. In contrast, for the high entropy composition (*x* = 0.3), complete domain switching was achieved under the DC‐bias field, as evident from the isotropic color distribution [Figure S3 (b_2_), Supporting Information]. Therefore, it is concluded that the *x* = 0.0 ceramic comprises micro‐/nano‐domains, while the *x* = 0.3 composition is dominated by PNRs. To deeply understand the reason behind the high energy storage in the optimal sample (*x* = 0.3), the scanning transmission electron microscopy (STEM) analysis is performed [**Figure** **3**a]. As given in Figure 3b, the lattice fringes calculated from the inset of Figure 3a are consistent with previously investigated results.^[^ ^28^ ^]^ No obvious domain structure is identified in this low‐resolution image. However, a weak contrast with small patches indicates the absence of long‐range ferroelectric order. A blotchy morphology with a 2–3 nm size should be related to the PNRs due to strong local structure heterogeneity.^[^ ^38^ ^]^ These PNRs actively responded to the externally applied field and generated ultralow dielectric/hysteresis losses, which are beneficial for energy storage performance.^[^ ^20^ ^]^

To further elucidate the local structure and nature of the PNRs from the perspective of cation displacement, the atomic‐resolution high‐angle annular dark‐field (HAADF) STEM image is captured along [100]_c_ axis [Figure 3c]. The 2D Gaussian function fitting is used to determine the accurate position of the A‐site and B‐site atomic columns. The A‐site atoms are larger and brighter, while the B‐site atoms are darker and smaller. The atomic displacement vectors are calculated from the offset of the A/B‐site atom center relative to its four neighboring B/A‐site atoms.^[^ ^23^, ^25^ ^]^ The RGB colors indicate the orientations, and the length of the vectors denotes the amplitude of the atomic displacement. In the [100]_c_ image, the coherent patches of 1–3 nm size with horizontal displacement vectors represent the PNRs of T phase. Here, some regions with almost zero polarization can be observed, which illustrate the C phase. However, the regions with [011]_c_ polarization vectors are either R or O phase. Further details of C, R/O, and T phases are provided in the magnified images in Figure 3d. The statistical distribution of R/O and T phases was calculated and is presented in Figure 3e. To further discriminate the R and O phases, the STEM‐HAADF image is taken along the [110]_c_ axis [see Figure 3f]. Now the horizontal polarization vector specifies symmetry of T phase and projections along [111]_c_ and [1$\bar{1}$0]_c_ directions signifying the R and O phases, respectively. The detailed and enlarged views for the PNRs of the R, O, and T symmetries are shown in Figure 3g. These detected multiphase R, O, T, and C clusters confirm the highly perturbed local structure of the *x* = 0.3 sample. Figure 3h indicates the statistical distribution of polarization vector calculated from the [110] HAADF STEM image. The scattered distribution of R, O, and T symmetries confirms the strong local fluctuation of polarization anisotropy.^[^ ^43^ ^]^ These multiphase PNRs ease polarization rotation, which leads to excellent energy storage performance. To further visualize the local distortion, the polarization angle and amplitude mappings were calculated from both [100]_c_ and [110]_c_ as shown in Figure 3i,j. Both polarization magnitude and polarization angle maps reveal highly disordered, randomly dispersed clusters. About 1–3 nm island‐like polar clusters are linked together by low‐symmetry interfaces, rather than being sequestered within the nonpolar matrix.^[^ ^43^ ^]^ During the external applied field, these randomly oriented multiphase PNRs delayed polarization saturation, which leads to highbreakdown field and improves energy storage.

**Figure 4:**
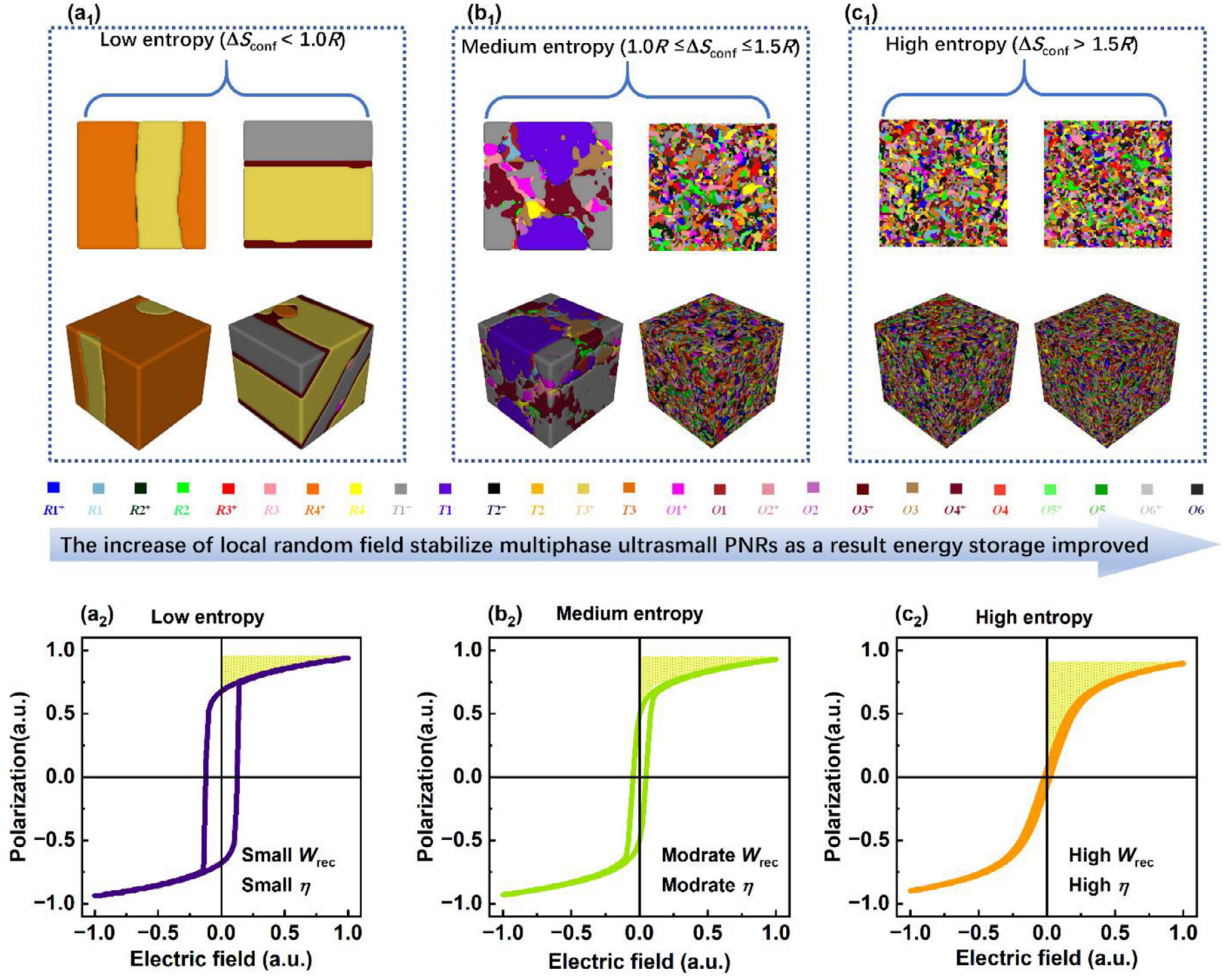
Phase field simulations. Phase field simulated a_1_–c_1_) 2D and 3D domain structures of BNBLT‐*x*NN system, where distinct colors represent various orientations of ferroelectric domains. a_2_–c_2_) Corresponding normalized *P*–*E* loops under the same electric field.

To validate the proposed mechanism for high‐energy storage performance, the theoretical phase field simulation was performed for the BNBLT‐*x*NN system. **Figure** **4** shows the 2D and 3D domain structures with varying length scale, where distinct colors represent the different polarization orientations and their corresponding calculated unipolar *P*–*E* loops. In ferroelectric, a composition designed near the MPB exhibited macroscopic symmetries, which are acknowledged for enhancing piezoelectric performance.^[^ ^44^, ^45^ ^]^ In contrast, relaxor ferroelectrics are composed of ultrasmall local polar symmetries embedded throughout a nonpolar cubic matrix.^[^ ^16^ ^]^ These polymorphic PNRs are randomly distributed, which delays polarization saturation, leading to high energy storage performance.^[^ ^27^ ^]^ Guided by preliminary experimental results, we developed a strategy to design a lead‐free relaxor ferroelectric exhibiting polymorphic PNRs. As evident from phase field simulated images in Figure 4 (a_1_), the low entropy ceramics Δ*S*_conf_ < 1.0*R*) possess the long‐range order and bimodal micro‐domains. Now the irreversible switching of these macroscopic domains induces a square shape *P*–*E* loop with large hysteresis loss, which shows poor energy storage performance as given in Figure 4 (a_2_). The ferroelectric loop calculated by phase field simulation is consistent with the experimental *P*–*E* loop as shown in Figure S1b (Supporting Information). Guided by Phase field simulation, the incorporation of NN and La‐donor doping into BNT‐based ceramics would induce local random field/stress due to the strong ferroelectric activity of La^3+^ and Nb^5+^ on A and B crystallographic sites, respectively.^[^ ^20^ ^]^ Phenomenological relation and phase field simulations indicate that chemical modification disrupts long‐range ferroelectric order, resulting in nanodomain formation and a consequent transition to the medium entropy regime [Figure 4 (b_1_); Figure S1a, Supporting Information]. The polymorphic nono‐domains minimize the energy barrier for domain switching due to the presence of various intermediate phases. Thus, the multiphase nano‐domains display a slimmer *P*–*E* loop, as shown in Figure 4 (b_2_). The medium‐entropy composition exhibited early saturation in its *P*–*E* loops, leading to a moderate energy storage performance (*W*_rec_ < 10 J cm^−3^ and *η* ≤ 80%). In this work, the *x* = 0.3 composition exhibited the highest high‐entropy (Δ*S*_conf_ = 1.72*R*) and contains ultrasmall polymorphic PNRs [Figure 4 (c_1_)]. The multiphase PNRs promptly respond to the external stimulus, thereby reducing the intrinsic loss of the polarization loops, as shown in Figure 4 (c_2_). The phase field simulations validate our experimental results and are also consistent with prior studies.^[^ ^20^, ^21^ ^]^ The typical ferroelectrics (*x* = 0.0) and relaxor ferroelectrics (*x* ≥ 0.1) can be characterized by sharp and diffused phase transitions in the dielectric property, as evident from Figure S4a–e (Supporting Information). Dielectric loss is also an important parameter, which is strongly related to hysteresis loss or efficiency.^[^ ^20^ ^]^ Among other compositions, the *x* = 0.3 ceramics showed the lowest tan*δ* ≈ 0.006 which is stable over a broad temperature range [see Figure S4f, Supporting Information]. According to Landau phenomenological theory, multiphase PNRs reduce polarization anisotropy and minimize dielectric loss.^[^ ^46^ ^]^ The chemical modifications induce domain miniaturization and reduce tangent loss. This effectively minimizes hysteresis loss, which is highly beneficial for enhancing energy storage. The SEM micrographs of the low‐entropy, medium‐entropy, and high‐entropy ceramics are shown in Figure S5 (a_1_–c_1_) (Supporting Information). Many studies have shown that *E*_B_ improves by reducing the average grain size.^[^ ^3^, ^17^, ^24^ ^]^ The low‐entropy BNT‐BT composition near the MPB exhibited a large average grain size of 2.33 µm. In contrast, the grain size decreased significantly to 0.95 µm for the medium‐entropy (*x* = 0.1) and 0.77 µm for the high‐entropy (*x* = 0.3) compositions. Phase field simulation strongly substantiates the logic behind the chemical composition design and also provides direct insight into the origin of the high energy storage performance.

**Figure 5:**
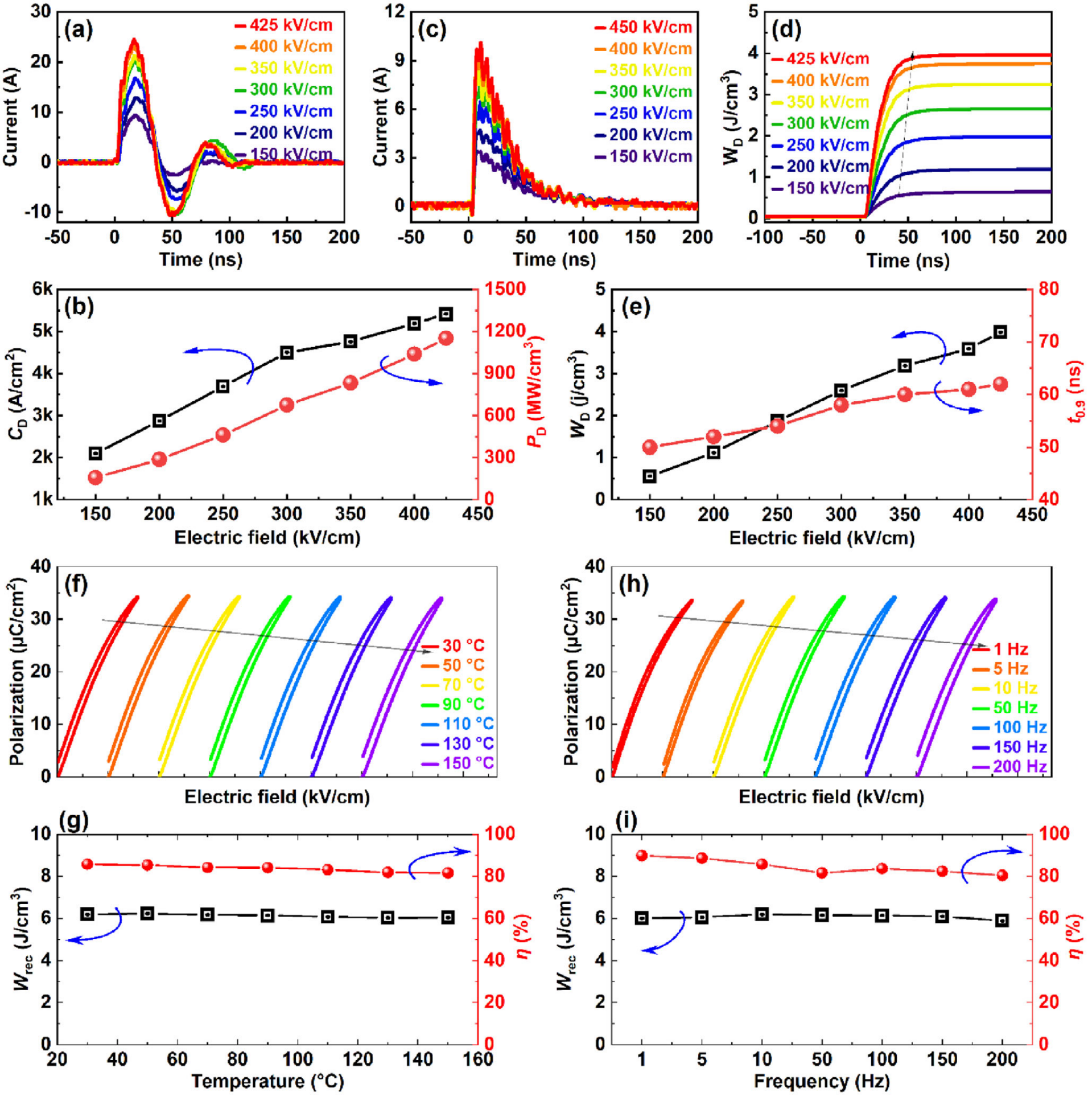
Charge–discharge property and energy storage stability: a) Underdamped current–time (*I*–*t*) curves at different applied field 150–425 kV cm^−1^, and b) *C*_D_ and *P*_D_ as a function of electric field. c) Overdamped *I*–*t* curves and d) time dependence *W*_d_ under the applied 150–425 kV cm^−1^. e) *W*_D_ and *t*_0.9_ at different applied electric fields. f–i) Temperature and Frequency‐dependent unipolar *P*–*E* loops with corresponding energy storage performance parameters.

For pulsed power capacitor technology, the high voltage direct current charge–discharge performance is also a key parameter. To discover the potential of the investigated materials for real device applications, the underdamped oscillating waveforms were measured a different applied fields from 150 to 425 kV cm^−1^ [**Figure** **5a**]. From these current–time (*I*–*t*) curves, the corresponding current density (*C*_D_ = *I*_ma_ *_x_*/*A*) and power density (*P*_D_ = *E*×*I*_ma_ *_x_*/2*A*) can be calculated, where *I*_ma_ *_x_*, *E*, and *A* represent the maximum current peak, applied field, and the electrode area, respectively.^[^ ^47^ ^]^ As shown in Figure 5b, both *C*_D_ and *P*_D_ increase monotonically with increasing applied field. The highest *C*_D_ ≈ 5416 A cm^−2^ and *P*_D_ ≈ 1150 MW cm^−3^ are realized under the applied field of 425 kV cm^−1^. This power density is much higher than that of prior studies.^[^ ^8^, ^21^, ^48^ ^]^ Figure 5c,d indicates the undamped current and discharge current density (*W*_D_), which can be calculated as: (*W*_D_ = *R*∫$I_{\left(t\right)}^{2}$ *dt*/*V* where *R, I*, and *V* represent the resistance, undamped current, and volume of the sample, respectively.^[^ ^22^, ^24^ ^]^ The *W*_D_ is gradually increased with increasing applied field and reaches the highest value of ≈ 4 J cm^−3^ at 425 kV cm^−1^. The *t*_0.9_ is also an important parameter, which indicates the time duration of a capacitor to release 90% of its stored energy. Additionally, *t*_0.9_ also slightly increased from 50 to 62 ns with increasing applied field from 150 to 425 kV cm^−1^ [Figure 5e].

For practical applications, thermal and frequency stability are crucial parameters for pratical applications. To evaluate this, we measured the unipolar *P*–*E* loops under the applied field of 470 kV cm^−1^ as a function of temperature and frequency. As shown in Figure 5f, a slim *P*–*E* hysteresis loop is consistently maintained over a broad temperature range 30–150 °C. A high *W*_rec_ ≈ 6.2 ± 0.15 J cm^−3^ and *η* ≈ 85.8 ± 4.1% were achieved, indicating the excellent thermal stability for high temperature applications in real devices [Figure 5g]. As shown in Figure S4f (Supporting Information), among the other compositions, the *x* = 0.3 sample exhibits a low and stable tan*δ* over a wide temperature range. This temperature‐insensitive energy storage is mainly related to the small dielectric loss and its thermal stability over a wide range.^[^ ^17^, ^20^ ^]^ The low dielectric loss minimizes hysteresis loss and prevents the device from heating during operation. Furthermore, frequency‐dependent unipolar *P*–*E* loops were tested at 470 kV cm^−1^ over the range of 1–200 Hz, as shown in Figure 5h. It can be seen that polarization response slightly varied over the diverse frequency range. In this work, the high and impressive frequency stable *W*_rec_ of 6.2 ± 0.2 J cm^−3^ and *η* of 85.8 ± 5.2%% over 1 to 200 Hz are promising results in the lead‐free ceramics [Figure 5i]. The ultrahigh energy storage performance, *W*_rec_ of 12.4 J cm^−3^, and *η* of 82.6% with good thermal/frequency stability of this work meets the requirements of advanced pulse power technology. The phase field simulations showed strong agreement with experimental observations, thereby validating the proposed mechanisms for the high energy storage performance.

## 3 Conclusion

In this work, we present a high‐entropy composition design strategy by a synergistic approach of donor‐doping and NN addition that manipulates the energy landscape of BNT‐based ceramics, leading to unprecedented energy storage capabilities. This strategy concurrently achieves multiple critical objectives for the improvement of energy storage performance. Both La‐doping and NaNbO_3_ induce intense local random field/stress, which disrupts the long‐range ferroelectric order and stabilizes ultrasmall polar nano‐regions. These multiphase PNRs reduce polar anisotropy and facilitate polarization reorientation; as a result, the overall energy storage improved. Donor doping also inhibits charge defects and suppresses dielectric/hysteresis losses, which are beneficial for energy storage. This effective approach yields a superior *W*_rec_ of 12.4 J cm^−^^3^ and *η* of ≈82.6% under the applied field of 790 kV cm^−1^. A high *W*_rec_ ≈ 6.2 ± 0.15 J cm^−3^ and *η* ≈ 85.8 ± 4.1%, maintaining over a broad temperature range 30–150 °C is highly beneficial for real device applications. Additionally, the outstanding *P*_D_ ≈ 1150 MW cm^−3^ alongside fast charge–discharge *t*_0.9_ ≈62 ns are also encouraging results. Thus, the proposed high‐entropy engineering strategy provides the transformative framework for the development of next‐generation dielectric capacitors.

## 4 Experimental Section

### Ceramics Fabrication

In this work, a new lead‐free high‐entropy system was designed according to the chemical formulae: (1‐*x*)(Bi_0.32_Na_0.32_Ba_0.32_La_0.04_)TiO_3_‐*x*NaNbO_3_ with *x* = 0.0, 0.1, 0.2, 0.3, and 0.4. The ceramics were synthesized using a conventional solid‐state reaction method. High‐purity Aladdin precursor powders of Na_2_CO_3_, Bi_2_O_3_, BaCO_3_, La_2_O_3_, TiO_2_, and Nb_2_O_5_ were mixed according to the target stoichiometry. The mixture was ball‐milled at 250 rev min^−1^ for 24 h, dried at 120 °C, and subsequently calcined at 850 °C for 5 h in air. The calcined powder was subjected to a second high‐speed (350 rev min^−1^) ball‐milling for 24 h. The resulting powder was then uniaxially pressed into 10 mm diameter pellets. These pellets were sintered in the temperature range of 1140–1200 °C for 2 h in air.

### Crystal Structure, Morphology, and Atomic Local Nanostructure Investigations

The crystal structure of the sintered ceramics was characterized by X‐ray diffraction (XRD; Rigaku MiniFlex 600, Japan). For the surface morphology investigation and elemental analysis, a scanning electron microscopy (SEM; S‐4800, Japan) was used. For domain morphology analysis, the ceramic pellets were polished to a thickness of ≈200 µm using diamond paste and examined by piezoresponse force microscopy (PFM; MFP‐3D Origin, Asylum Research, USA). Atomic‐scale imaging was performed using high‐angle annular dark‐field scanning transmission electron microscopy (HAADF‐STEM) on an aberration‐corrected FEI Titan Cubed Themis G2 300 microscope. The polarization vectors of magnitude and angle were calculated by a custom MATLAB scripts software.

### Dielectric Property

Temperature‐dependent dielectric properties were measured using an impedance analyzer (Keysight E4990A), and impedance analysis was carried out using an impedance spectroscopy (GWDS‐003, TG, GERMANY).

### Energy Storage Property

For electrical characterization, the sintered pellets were polished to a thickness of 50–80 µm. A continuous gold electrode was deposited on one side to serve as the bottom electrode, while an array of small point electrodes (area ≈ 0.8 mm^2^) was deposited on the opposite side. Polarization‐electric field (*P*–*E*) hysteresis loops were measured using a ferroelectric tester (Radiant Technologies, USA). The charge–discharge performance was evaluated under both undamped and overdamped conditions using a commercial tester (Tongguo Technology CFD‐003, China).

## Conflict of Interest

The authors declare no conflict of interest.

## Supporting information



Supporting Information

## Data Availability

The data that support the findings of this study are available from the corresponding author upon reasonable request.
