# Supplementary material for: High‐Entropy Chemical Composition Design for Ultrahigh Capacitive Energy Storage
Source: Adv Sci (Weinh). 2025 Dec 3;13(9):e21163. doi: 10.1002/advs.202521163 (PMC12904035; doi:10.1002/advs.202521163)
Supplement: Supplementary file 1 — Supporting Information [file ADVS-13-e21163-s001.docx]

**High-entropy chemical composition design for ultrahigh capacitive energy storage**

Muhammad Habib ^1^, Haoyu Wang ^2^, Weisan Fang ^2^, Attaur Rahman ^3^, Maqbool Ur Rehman ^3^, Ting Wang ^1^, Qingfeng Zhu ^1^, Muhammad Javid Iqbal ^4^, Xiaoming Shi ^2, *^, He Qi ^5,^ ^*^, Weiping Gong ^1,^ ^*^

^1^ *Guangdong Provincial Key Laboratory of Electronic Functional Materials and Devices, Huizhou University, Huizhou, Guangdong 516001, China*

^2^ *School of Mathematics and Physics, University of Science and Technology Beijing, Beijing 100083, China*

^3^ *School of Materials Science and Engineering, Anhui Polytechnic University, Wuhu 241000, China*

^4^ *Department of Physics, University of Peshawar, Peshawar, 25120, Pakistan*

^5^ *School of Materials Science and Engineering, Hainan University, Haikou, Hainan 57228, China*

*Corresponding authors: [shiming_870@163.com](mailto:shiming_870@163.com); [qihe@hainanu.edu.cn](mailto:qihe@hainanu.edu.cn); [gwp@hzu.edu.cn](mailto:gwp@hzu.edu.cn)

**Supporting Information**

High entropy is an emerging idea for improved energy storage properties. For high-entropy compositions design the inhomogeneity can be quantified according to the following formula;

*S*_conf_ = -*R*[$\sum_{i}^{n} x_{i}{lnx}_{i}$ + $\sum_{i}^{m} x_{k}{lnx}_{k}$] where ‘*n*’ and ‘*m*’ are the atomic species, while *i* and *k* represent the atomic contents of A-site and B-site cations, respectively. For the MPB composition 0.94(Bi_0.5_Na_0.5_)NbO_3_-0.06BaTiO_3_ (BNT-6BT), the entropy configuration is *S*_conf_ = 0.878*R* as given in Table S1*.* The configurational entropy (S_conf_) for the (1-*x*)(Bi_0.32_Na_0.32_Ba_0.32_La_0.04_)TiO_3_-*x*NaNbO_3_ system was calculated for compositions *x* = 0.0, 0.1, 0.2, 0.3, 0.4, and 0.5 (Tables S2-S7).

*S*_conf_ = -*R*[Na×ln(Na)+Bi×ln(Bi) )+Ba×ln(Ba) + La×ln(La) +Ti×ln(Ti) +Nb×ln(Nb)]

**Table S1: Entropy calculation for BNT-6BT composition.**

| Atoms | Ratio | %age | ln(*x*) | *x*ln(*x*) |
| --- | --- | --- | --- | --- |
| Na | 0.47 | 0.47 | -0.75502 | -0.35486 |
| Bi | 0.47 | 0.47 | -0.75502 | -0.35486 |
| Ba | 0.06 | 0.06 | -2.81341 | -0.1688 |
| Ti | 1 | 1 | 0 | 0 |
|  | 2 |  |  | -0.8785 |
|  |  |  |  |  |

**Table S2: Entropy calculation for *x* = 0.0 composition.**

| Atoms | Ratio | %age | ln(*x*) | *x*ln(*x*) |
| --- | --- | --- | --- | --- |
| Na | 0.32 | 0.32 | -0.646 | -0.339 |
| Bi | 0.32 | 0.32 | -1.496 | -0.335 |
| Ba | 0.32 | 0.32 | -1.496 | -0.335 |
| La | 0.04 | 0.04 | -3.575 | -0.100 |
| Ti | 1 | 1 | 0 | 0 |
|  | 2 |  |  | -1.222 |

**Table S3: Entropy calculation for *x* = 0.1 composition.**

| Atoms | Ratio | %age | ln(*x*) | *x*ln(*x*) |
| --- | --- | --- | --- | --- |
| Na | 0.32 | 0.397 | -0.924 | -0.367 |
| Bi | 0.32 | 0.297 | -1.214 | -0.361 |
| Ba | 0.32 | 0.297 | -1.214 | -0.361 |
| La | 0.04 | 0.009 | -4.711 | -0.042 |
| Ti | 0.9 | 0.9 | -0.105 | -0.095 |
| Nb | 0.1 | 0.1 | -2.303 | -0.230 |
|  | 2 |  |  | -1.455 |

**Table S4: Entropy calculation for *x* = 0.2 composition.**

| Atoms | Ratio | %age | ln(*x*) | *x*ln(*x*) |
| --- | --- | --- | --- | --- |
| Na | 0.32 | 0.456 | -0.785 | -0.358 |
| Bi | 0.32 | 0.256 | -1.362 | -0.349 |
| Ba | 0.32 | 0.256 | -1.362 | -0.349 |
| La | 0.04 | 0.032 | -3.442 | -0.1101 |
| Ti | 0.8 | 0.8 | -0.223 | -0.178 |
| Nb | 0.2 | 0.2 | -1.609 | -0.322 |
|  | 2 |  |  | -1.660 |

**Table S5: Entropy calculation for *x* = 0.3 composition.**

| Atoms | Ratio | %age | ln(*x*) | *x*ln(*x*) |
| --- | --- | --- | --- | --- |
| Na | 0.32 | 0.524 | -0.646 | -0.339 |
| Bi | 0.32 | 0.224 | -1.496 | -0.335 |
| Ba | 0.32 | 0.224 | -1.496 | -0.335 |
| La | 0.04 | 0.028 | -3.575 | -0.100 |
| Ti | 0.7 | 0.7 | -0.357 | -0.250 |
| Nb | 0.3 | 0.3 | -1.204 | -0.361 |
|  | 2 |  |  | -1.720 |

**Table S6: Entropy calculation for *x* = 0.4 composition.**

| Atoms | Ratio | %age | ln(*x*) | *x*ln(*x*) |
| --- | --- | --- | --- | --- |
| Na | 0.32 | 0.592 | -0.524 | -0.310 |
| Bi | 0.32 | 0.192 | -1.650 | -0.317 |
| Ba | 0.32 | 0.192 | -1.650 | -0.317 |
| La | 0.04 | 0.024 | -3.730 | -0.090 |
| Ti | 0.6 | 0.6 | -0.511 | -0.306 |
| Nb | 0.4 | 0.4 | -0.916 | -0.366 |
|  | 2 |  |  | -1.710 |

**Table S7: Entropy calculation for *x* = 0.5 composition.**

| Atoms | Ratio | %age | ln(*x*) | *x*ln(*x*) |
| --- | --- | --- | --- | --- |
| Na | 0.32 | 0.66 | -0.416 | -0.2742 |
| Bi | 0.32 | 0.16 | -1.832 | -0.293 |
| Ba | 0.32 | 0.16 | -1.832 | -0.293 |
| La | 0.04 | 0.02 | -3.912 | -0.078 |
| Ti | 0.5 | 0.5 | -0.693 | -0.346 |
| Nb | 0.5 | 0.5 | -0.693 | -0.346 |
|  | 2 |  |  | -1.632 |

The results indicate that *S*_conf_ reaches a maximum at *x* = 0.3 and then decreases for higher NN (*x* > 0.3) content. This entropy trend can be understood by analyzing the site occupancy. In this system, the A-site entropy is dominant due to the mixing of four different cations (Na⁺, Bi³⁺, Ba²⁺, La³⁺), compared to only two (for *x* > 0.0) on the B-site (Ti⁴⁺, Nb⁵⁺). The initial composition (*x* = 0.0) has an equal atomic ratio of Bi:Na:Ba (32:32:32) on the A-site, leading to a high initial entropy. However, as NaNbO₃ (NN) is added, it introduces additional Na⁺ onto the A-site and Nb⁵⁺ onto the B-site. The key point is that the Na⁺ concentration on the A-site increases disproportionately. At *x* = 0.3, the A-site cation ratio becomes approximately Na:Bi:Ba:La ⸬ 52:22:22:4. As the Na⁺ fraction surpasses 50%, the system begins to deviate from an ideal, maximally random mixture of multiple cations. With further increase in NN content (*x* > 0.3), the A-site becomes increasingly dominated by a single cation (Na⁺), which reduces the configurational entropy. Therefore, *x* = 0.3 represents the composition near the entropy maximum in this series. Since high configurational entropy is known to enhance phase stability and disrupt long-range ferroelectric order, favoring the relaxor behavior desirable for energy storage, this composition (*x* = 0.3) is optimized for the best energy storage performance.

**
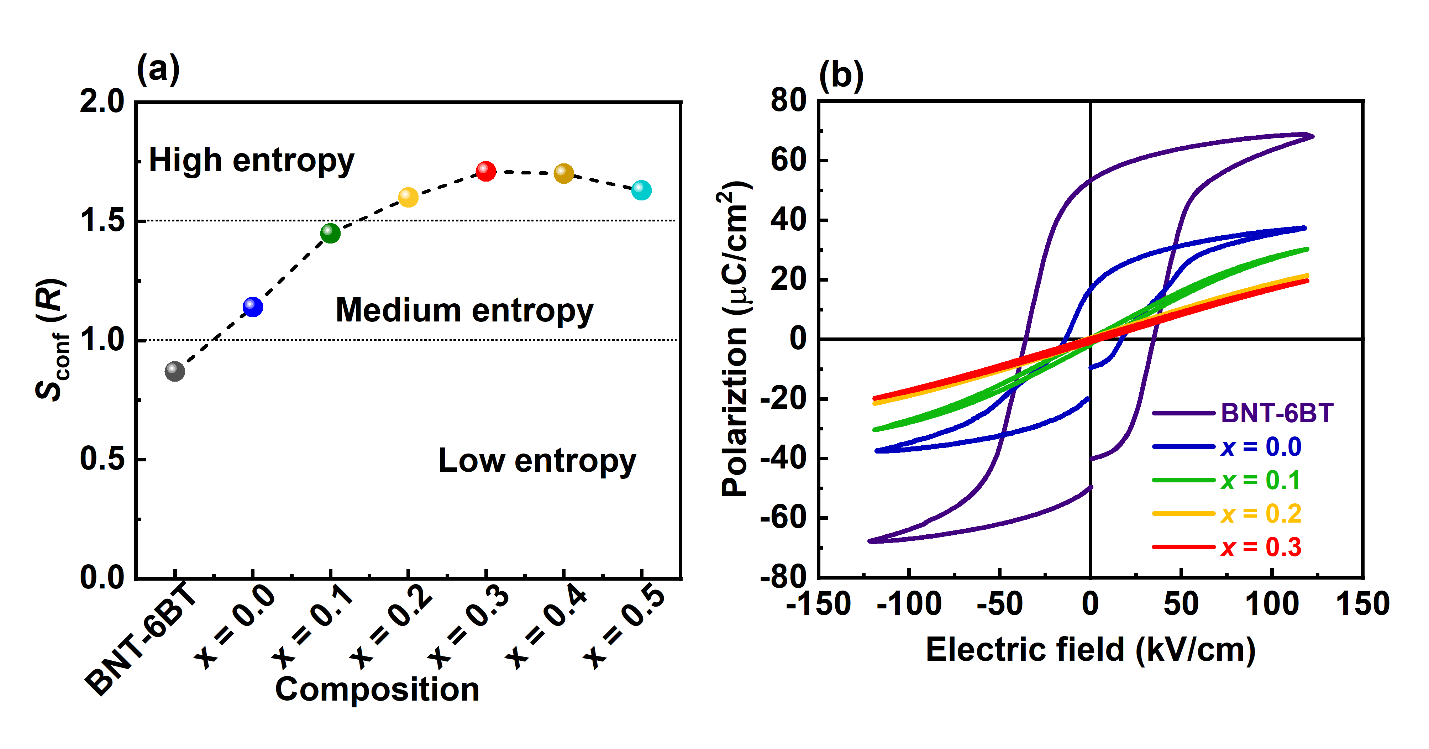
**

**Figure S1:** (a) Entropy configuration for BNT-6BT (low entropy composition near the MPB) and BNBLT-*x*NN (*x* = 0.0, 0.1, 0.2, 0.3, 0.4, and 0.5) ceramics system. (b) Bipolar *P*-*E* loops at 120 kV/cm for BNT-6BT and *x* = 0.0, 0.1, 0.2, and 0.3 ceramics.

**Phase-field simulation**

We employed a phase field modeling approach to simulate the evolution of ferroelectric domain structures as a function of composition. The domain patterns are characterized by the spatial distribution of the spontaneous polarization vector, *P* (*P*_1_, *P*_2_, *P*_3_). We simulate the temporal evolution of polarization by solving the time-dependent Ginzburg-Landau (TDGL) equation, concurrently addressing the stress and electric equilibrium equations to obtain the resulting displacement and electric displacement fields ^[1]^.

$$\begin{aligned} \frac{\partial P_{i}}{\partial t}=-L\frac{\delta F}{\delta P_{i}}+E_{i}^{thermal}\#\left( 1 \right) \end{aligned}$$

$$\begin{aligned} \frac{\partial}{\partial x_{j}}\left( \sigma_{ij}\left( r,t \right) \right)=0\#\left( 2 \right) \end{aligned}$$

$$\begin{aligned} \nabla\cdot D=\rho_{f}\#\left( 3 \right) \end{aligned}$$

The polarization evolution is governed by the time-dependent Ginzburg-Landau equation, $\frac{\delta F}{\delta P_{j}}$, where *L* is a kinetic coefficient related to domain wall mobility. *F* shows the total free energy of the system. *σ*_ij_ , $\rho_{f}$, *r,* and *t* indicate the stress tensor, free charge density, the spatial coordinate, and time, respectively. Now the total free energy of a bulk system can be defined as:

$$\begin{aligned} F=F_{\mathrm{bulk}}\left( \boldsymbol{P} \right)+F_{\mathrm{grad}}\left( \boldsymbol{P} \right)+F_{\mathrm{elastic}}\left( \boldsymbol{P} \right)+F_{\mathrm{elec}}\left( \boldsymbol{P,E} \right) \\ =\int_{V} \left( f_{\mathrm{bulk}}+f_{\mathrm{grad}}+f_{\mathrm{elastic}}+f_{\mathrm{elec}} \right)dV\#\left( 4 \right) \end{aligned}$$

Here, *F* integrates contributions from bulk free energy$F_{\mathrm{bulk}}(\boldsymbol{P})$, domain-wall energy $F_{\mathrm{grad}}(\boldsymbol{P})$, elastic energy $F_{\mathrm{elastic}}(\boldsymbol{P})$, and electrostatic energy $F_{\mathrm{elec}}(\boldsymbol{P,E})$, where ***E*** is the applied static electric field, $f_{\mathrm{bulk}}$, $f_{\mathrm{grad}}$, $f_{\mathrm{elastic}}$ and $f_{\mathrm{elec}}$ are corresponding energy densities. The bulk free-energy density is expressed as an eight-order polynomial expansion:

$$f_{bulk}=\alpha_{1}\left( P_{1}^{2}+P_{2}^{2}+P_{3}^{2} \right)+\alpha_{11}\left( P_{1}^{4}+P_{2}^{4}+P_{3}^{4} \right)+\alpha_{12}\left( P_{1}^{2}P_{2}^{2}+P_{1}^{2}P_{3}^{2}+P_{2}^{2}P_{3}^{2} \right)+\alpha_{111}\left( P_{1}^{6}+P_{2}^{6}+P_{3}^{6} \right)+\alpha_{112}\left[ P_{1}^{4}\left( P_{2}^{2}+P_{3}^{2} \right)+P_{3}^{4}\left( P_{2}^{2}+P_{1}^{2} \right)+P_{2}^{4}\left( P_{3}^{2}+P_{1}^{2} \right) \right]+\alpha_{123}P_{1}^{2}P_{2}^{2}P_{3}^{2} (5)$$

where $\alpha_{i}$, $\alpha_{ij}$, $\alpha_{ijk}$ are the Landau parameters. The elastic energy density can be written as $f_{\mathrm{elastic}}=\frac{1}{2}c_{ijkl}\left( \varepsilon_{ij}-\varepsilon_{ij}^{0} \right)\left( \varepsilon_{kl}-\varepsilon_{kl}^{0} \right)$, where $c_{ijkl}$ is the elastic stiffness tensor, $\varepsilon_{ij}$ is the total strain and $\varepsilon_{ij}^{0}$ is the eigenstrain. The eigenstrain can be described as $\varepsilon_{ij}^{0}=Q_{ijkl}P_{k}P_{l}$, where $Q_{ijkl}$ is the electrostrictive coefficient. The gradient energy density can be obtained by $f_{grad}=\frac{1}{2}{G_{ijkl}P}_{i, j}P_{k, l}$, where $G_{ijkl}$ is the gradient coefficient.

The electrostatic energy can be expressed as $f_{elec}=-P_{i}{\left( r \right)(E}_{i}\left( r \right)-{\frac{1}{2}P}_{i}(r)E_{i}^{in}(r)$, where $E_{i}^{in}(r)$is the *E*-field induced by the dipole moments, $E_{i}(r)$ is the applied electric field.

The simulation scale is 64d*x*×64dy×64d*z*, and the grid scales *dx* *d*y and *dz* are 1 nm. The Landau parameters of the Na_0.32_Bi_0.32_Ba_0.32_La_0.04_TiO_3_ system are, $\alpha_{1}$ = 3.34×10^5^×(*T*-381) C^-2^ m^2^ N, $\alpha_{11}$ = (4.69×T-20.46)×10^8^ C^-4^ m^6^ N, $\alpha_{12}$ = 3.23×10^8^ C^-4^ m^6^ N, $\alpha_{111}$ = (24.45-5.52×T)×10^9^ C^-6^ m^10^ N, $\alpha_{112}$ = 4.47×10^9^ C^-6^ m^10^ N, $\alpha_{123}$ = 4.91×10^9^ C^-6^ m^10^ N, *Q*_11_ = 0.1104 C^-2^ m^4^, *Q*_12_ = -0.0452 C^-2^ m^4^, *Q*_44_ = 0.0578 C^-2^ m^4^, *c*_11_ = 5.1×10^12^ N m^-2^, *c*_12_ = -2×10^11^ N m^-2^, *c*_44_ = 2×10^11^ N m^-2^, where *T* denotes the absolute temperature. To better align with experimental conditions, we introduced a Gaussian-distributed electric field whose magnitude scales with the entropy-dependent diffusion doping level. The field has a mean of 0 MV/cm and a standard deviation of *x* MV/cm, where *x* is a function of the configurational entropy.

**Crystal structure**

To correlate the energy storage performance of this work with phase structure, the XRD patterns were examined in the 2*θ* range of 10-80° [**Figure S2(a**)]. Generally, the (111), (100)/(200), and (110)/(220) peaks in the XRD are used as fingerprints for the identification of the R, T, and O symmetries, respectively. As evident from the enlarged view in **Figure S2(b)**, the *x* = 0.0 sample demonstrates an additional hum peak on the right-hand side of (200)_c_ and (220)_c_ peaks, which is an evident feature of T and O phases. This hump peak gradually disappears for the higher order of NN content (*x* > 0.1), confirming the emergence of the pseudo-cubic phase. Furthermore, an additional peak on the right side of the (220) peak near 2*θ* ≈ 67° indicates Cu kα_2_ radiation, which becomes more prominent in the relaxor ferroelectric ceramics ^[2]^. The XRD results indicate the crystal structure transforms from the long-range ferroelectric order to the short-range relaxor phase. Hence, the XRD results are consistent with ferroelectric response.

**
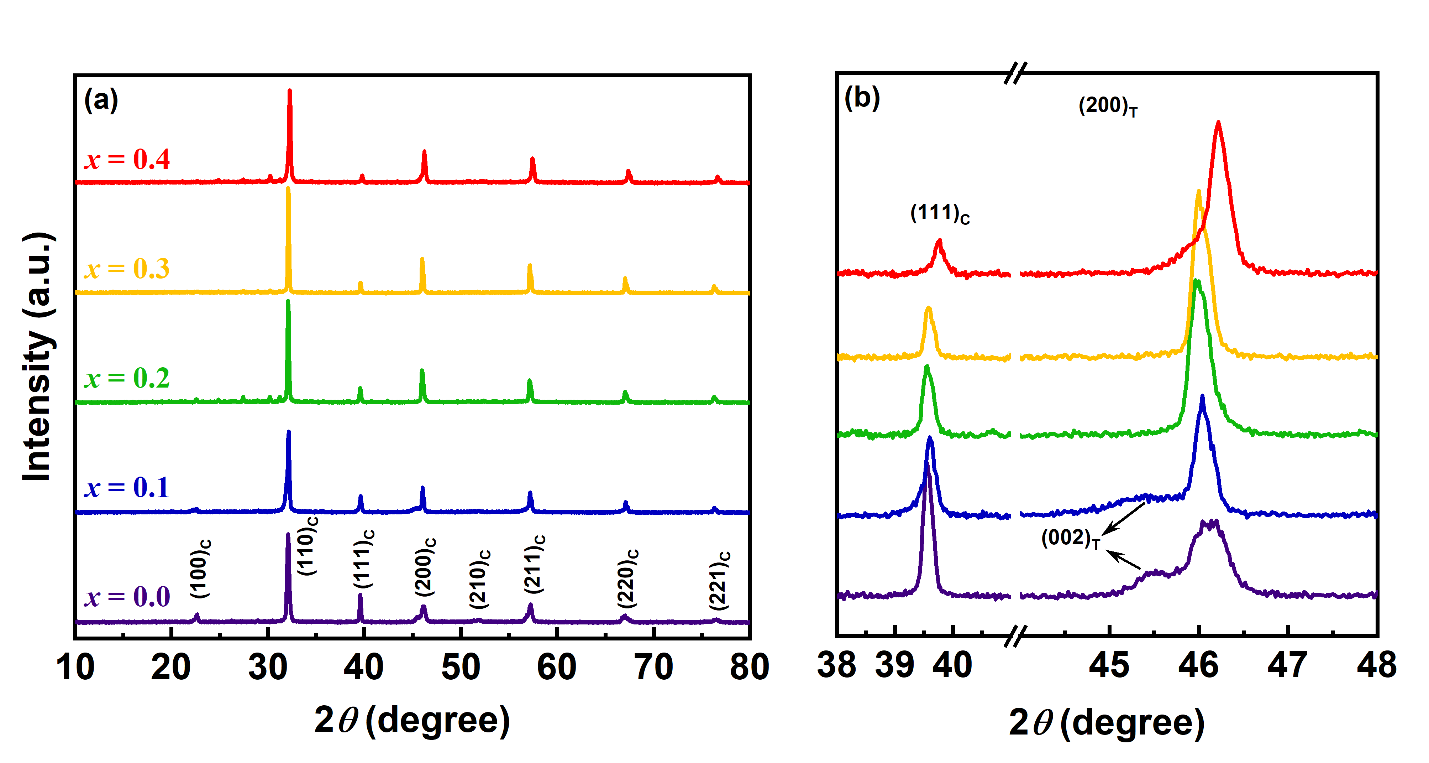
**

**Figure S2.** (a) XRD patterns for BNBLT-*x*NN system with *x* = 0.0, 0.1, 0.2, 0.3, and 0.4 ceramics. (b) Magnified view for the (111) and (200) peaks.

Piezoresponse Force Microscopy (PFM) measurements were conducted to probe domain dynamic behavior. It provides direct evidence of the nanoscale polar behavior. Figure S3(a_1_,b_1_) shows the domain structure of the virgin state for *x* = 0.0 and 0.3 ceramics. For *x* = 0.0 ceramic, the PFM phase images under DC bias field (+20 V and -20 V) show diverse color distributions. This indicates the presence of stable micro- and nano-domains that are only partially switched, consistent with larger, more rigid domain structures. In contrast, the high-entropy composition (*x* = 0.3), shows an isotropic color distribution that is completely switched by the DC bias field. This is a characteristic signature of ultrasmall, weakly correlated, and highly responsive PNRs, which aligns perfectly with the ultrasmall PNRs observed in our HAADF-STEM analysis.

**
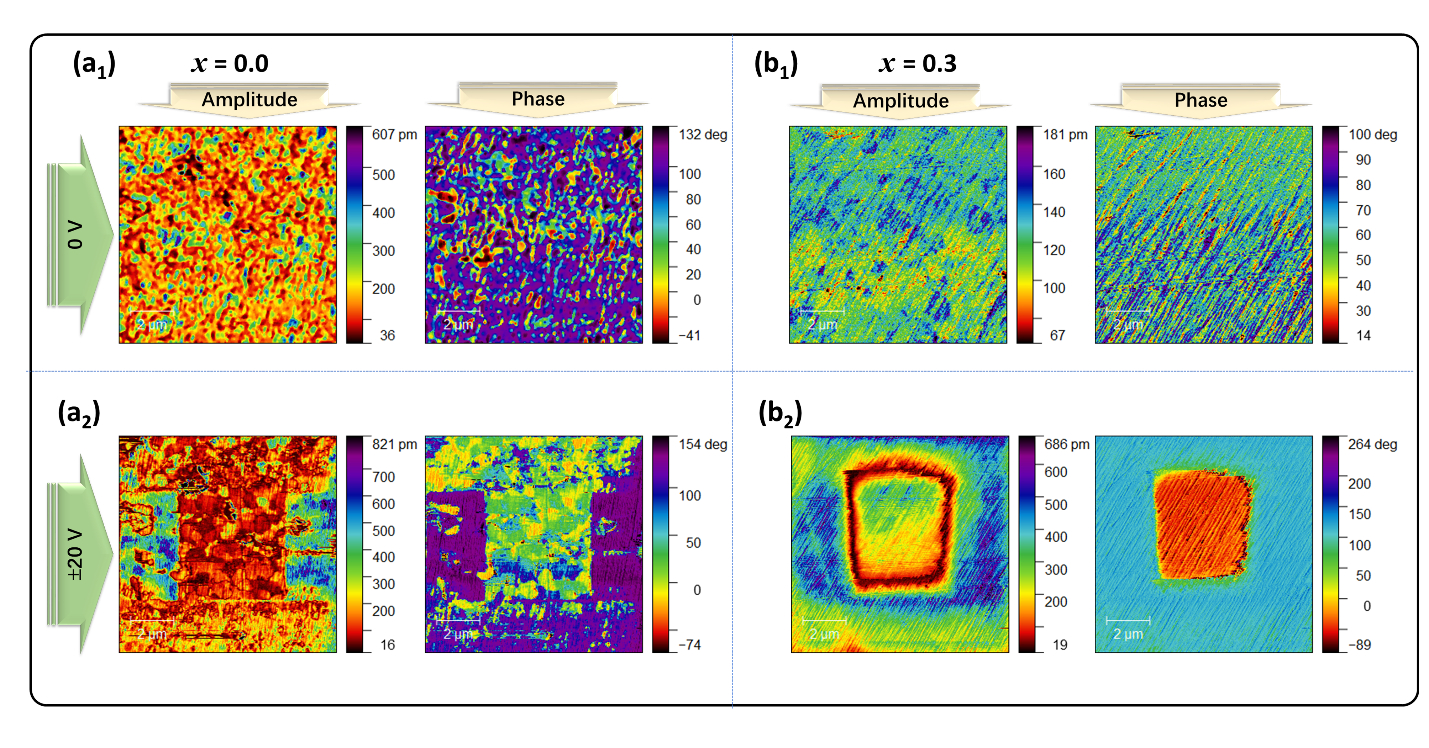
**

**Figure S3.** PFM amplitude and phase images of virgin surface at 0 V and under ±20 V for (a_1_,a_2_) *x* = 0.0 and (b_1_,b_2_) *x* = 0.3 ceramics.

To further comprehensively assess the relaxor ferroelectric characteristics of the design system, temperature-dependent dielectric properties were examined in the temperature range 25 to 400 °C at frequencies 100 Hz to 1 MHz [**Figure S3(a-e),** Supporting Information]. The *x* = 0.0 ceramic has a unique frequency-dependent dispersion and a high maximum temperature (*T*_m_ ≈ 230 °C). It can be observed that with increasing *x,* the *T*_m_ is gradually shifted towards the lower temperature. The NN addition in BNBLT disrupts the long-range order and stabilizes the short-range relaxor order, which dilutes the mutual coupling of PNRs; as a result, *T*_m_ shifts towards the lower temperature ^[3]^. The *x* = 0.3 ceramic presented a highly stable and frequency-independent dielectric constant over the whole temperature range, predicting high energy storage performance. For *x* = 0.4 sample dielectric constant suddenly increases above 300 °C at low frequencies, which is usually related to defect space charge conduction. Dielectric loss is also an important factor for high-energy storage. As shown in **Figure S3(f),** the medium entropy compositions (*x* = 0.0 and 0.1) displayed high tan*δ* ≈ 0.08 at room temperature. On the other hand, the high entropy composition (*x* = 0.3 and 0.4), the tan*δ* dramatically decreases to 0.005 and also remains stable over a wide temperature range. However, for *x* = 0.4 sample a sudden increase in tan*δ* value above 300 °C may be related to some defect charges. Hence, the ultralow dielectric of the optimal sample (*x* = 0.3) generates very small heat during polarization rotation under the externally applied field, leading to high breakdown strength ^[1]^.

**
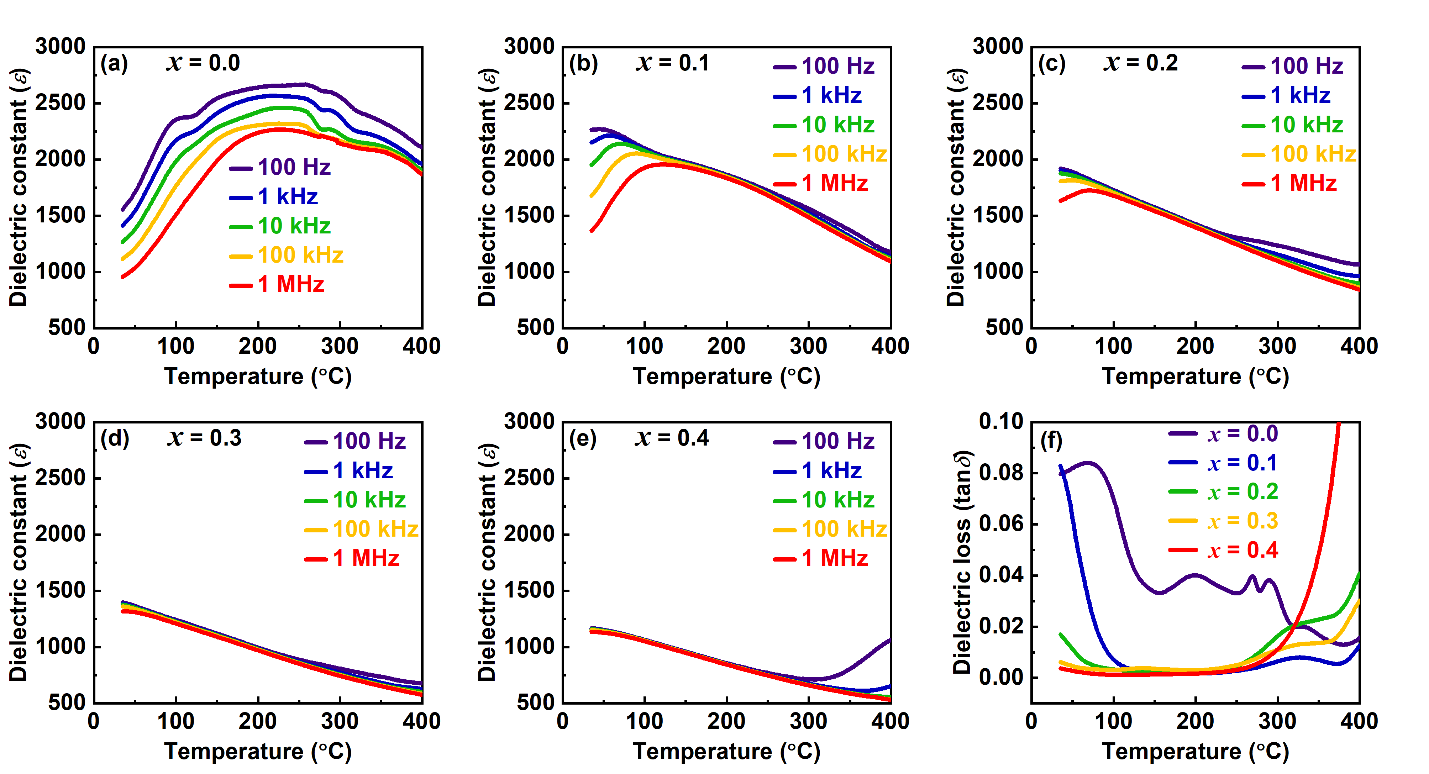
**

**Figure S4.** Temperature dielectric constant at 100 Hz, 1 kHz, 10 kHz, 100 kHz, and 1 MHz for (a) *x* = 0.0, (b) *x* = 0.1, (c) *x* = 0.2, (d) *x* = 0.3, (e) *x* = 0.4, and (f) dielectric loss at 10 kHz.

Reducing the average grain size in bulk ceramics significantly enhances the dielectric breakdown strength (*E*_b_) ^[4]^. Finer grains create a higher density of grain boundaries, which provide scattering sites for charge carriers and impede the propagation of the electrical breakdown path ^[5]^. Consequently, a more uniform microstructure with fine grains can withstand a much higher electric field before failure. In this work, the low-entropy BNT-BT composition near the morphotrophic phase boundary (MPB) exhibited a coarse microstructure distributed over a wide range with a large average grain size of 2.33 μm, as given in **Figure S5**(a_1_). In contrast, increasing the configurational entropy led to a significant grain refinement as a result average grain size reduced to 0.97 μm and 0.77 μm for the medium-entropy (*x* = 0.1) and high-entropy (*x* = 0.3) composites, respectively [**Figure S5(b_1_, c_1_)**]. Energy dispersive X-ray spectroscopy (EDS) elemental mapping confirmed the successful incorporation of all constituent cations, for low, medium, and high entropy composites [**Figure S5(a_2_-c_2_)**]. EDS analysis confirms the uniform distribution of all constituent elements, with no detectable segregation within the ceramic matrix.


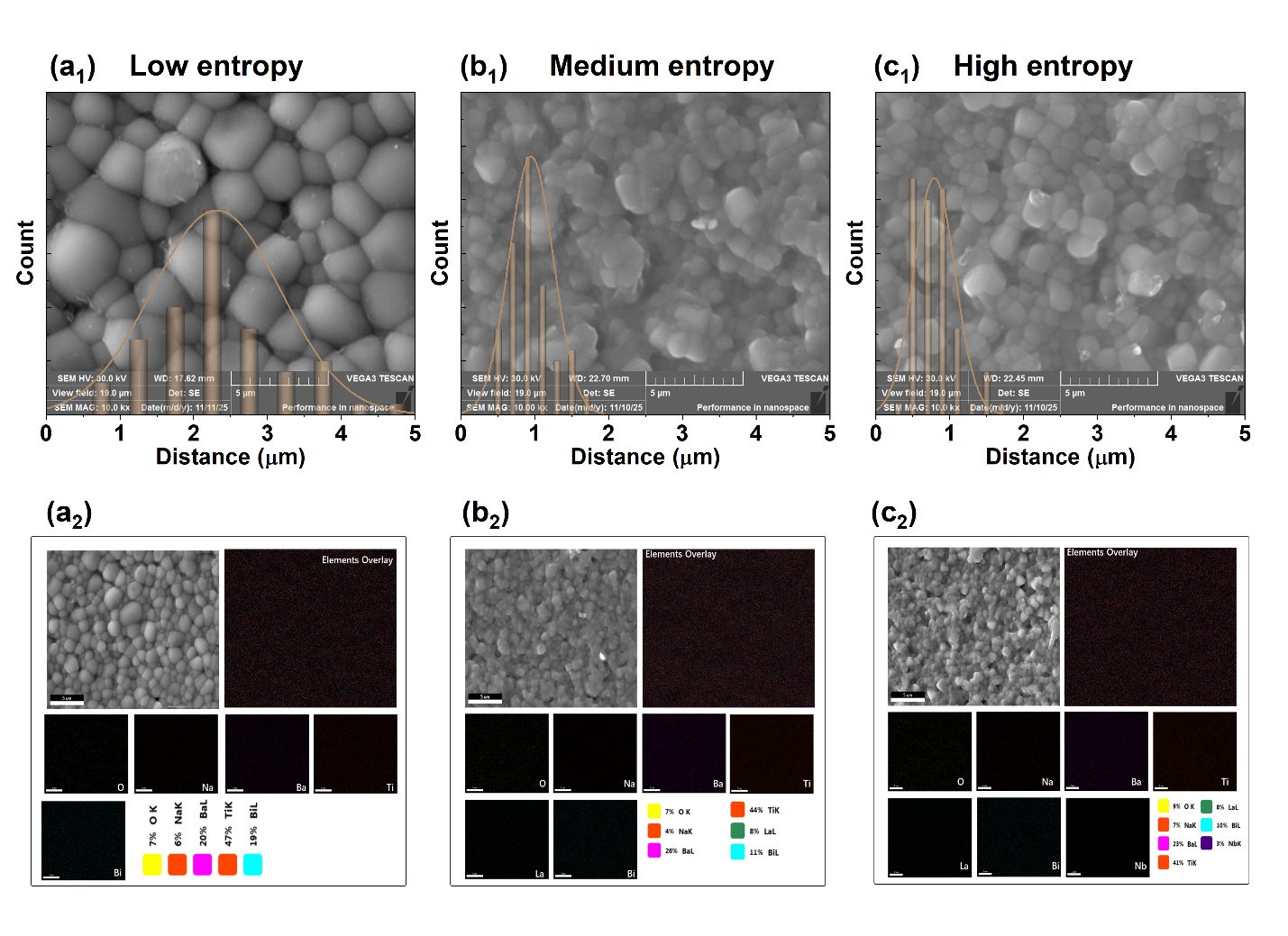


**Figure S5.** SEM and EDS analysis for (a_1,_ a_2_) BNT-BT design near the MPB and for BNBLT-*x*BT system (b_1_, b_2_) *x* = 0.0 and (c_1_, c_2_) *x* = 0.3 samples.

To deconvolute the resistivity change with La and NaNbO_3_ contents of the investigated ceramics. Three compositions, Bi_0.33_Na_0.33_Ba_0.33_La_0.01_TiO_3_ [*x* = 0.0 (La1)], Bi_0.32_Na_0.32_Ba_0.32_La_0.04_TiO_3_ [*x* = 0.0 (La4)], and 0.7Bi_0.32_Na_0.32_Ba_0.32_La_0.04_TiO_3_-0.3NaNBO_3_ [*x* = 0.3 (La4)] were chosen for the case study as given in Figure S6. The impedance spectroscopy revealed a distinct evolution in the electrical response with La-donor doping and NN content. The high radius of the semicircle of *x* = 0.0 (La4)as compared to the *x* = 0.0 (La1) ceramics is a clear indication of an increase in resistive behavior for the higher order of La-donor content [**Figure S6(a,b)**]. In the inset of **Figure S6(a)**, *R*_g_ (*C*_g_) and *R*_gb_ (*C*_g_) denote grain and grain boundary resistances (capacitances), respectively. For La4-3NN, another small arc indicates the grain boundary effect as evident from **Figure S6(c)**. The complex electric modulus and complex impedance are defined as *M*^*^ = *M*′ + *jM*′′ and *Z*^*^ = *Z*′ + *jZ*′′ where *M*′′ and *Z*′′ are the imaginary parts ^[6]^. In polycrystalline ceramics, the electrical modulus (*M*′′) and impedance (*Z*′′) provide information about the grains and grain boundary, respectively ^[4]^. From the normalized *Z*′′ and *M*′′ plots a large gap with Δ*f* = 7328 Hz for *x* = 0.0 (La1) indicate electrical non-uniformity within the ceramic. Specifically, a broad peak of *M*′′ related to the interfacial polarization response. However, for *x* = 0.0 (L4) and *x* = 0.3 (L4) samples the maximum frequency (*f*_max_) of *Z*′′ and *M*′′ overlapped, suggesting a more uniform electric field inside the ceramics. It emphasizes that the decrease in the gap of *Z*′′ and *M*′′ spectroscopic plot indicating suppression of interfacial polarization ^[4]^. In the high entropy composition [*x* = 0.3 (L4)], the two circuits become a single R-C circuit, suppressing interfacial polarization, resulting in high *E*_b_. Similar results have been observed in prior work ^[6, 7]^. Figure S6(g,h) shows the Nyquist and Z′′-*f* plots at the same temperature for comparison. The increase in the semicircle radius and shift *f*_max_ peak towards the higher frequency with the increase of La and NN content ascribed the improvement of electrical resistivity. More importantly, the electrical conductivity calculated from Jonscher’s power law further supports the increase in electrical resistivity for the 4 mol% of La content as compared with 1 mol%. From impedance analysis, it is evident that with the incorporation of both La and NN, increase in electrical resistivity. However, only 4% of the La-donor effect is more prominent than the 30% of NN content.


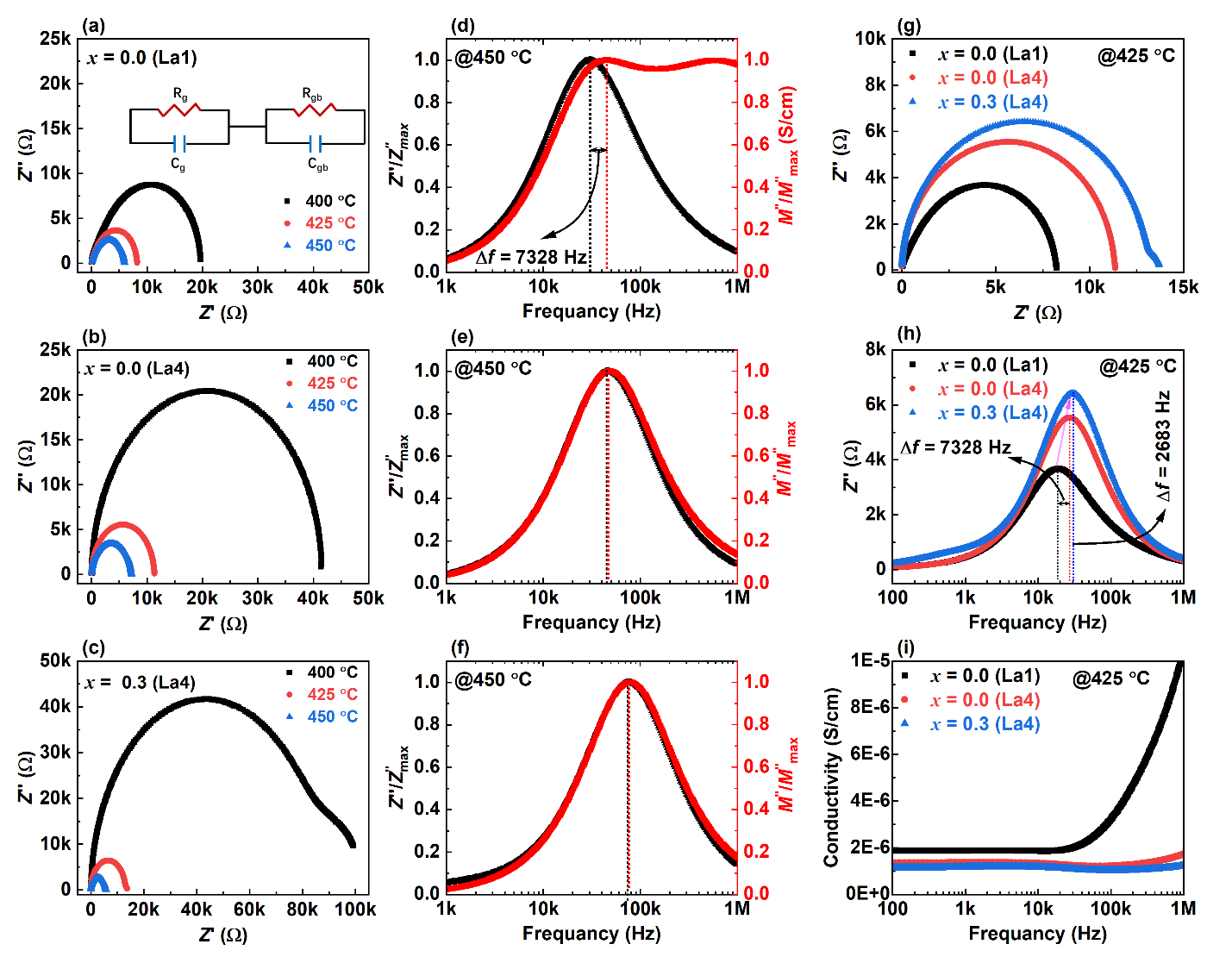


**Figure S6.** Complex impedance Nyquist plot at 400 °C, 425 °C, and 450 °C for (a) *x* = 0.0 (La1), (b) *x* = 0.0 (La4), and (c) *x* = 0.3 (La1) samples and (d-f) their corresponding spectroscopic plot *Z*^′′^/Z^′′^_max_ and *M*^′′^/ *M*^′′^_max_ as a function of frequency measured at 450 °C. (g) Comparison of Nyquist plot, (h) impedance, and (i) conductivity as a function of frequency at 425 °C.

**References**

[1] L. Chen, T. Hu, X. Shi, H. Yu, H. Zhang, J. Wu, Z. Fu, H. Qi, J. Chen, *Adv. Mater.* **2024**, 36, 2313285.

[2] M. Habib, L. Tang, G. Xue, A. Rahman, M.-H. Kim, S. Lee, X. Zhou, Y. Zhang, D. Zhang, *Chem. Eng. J.* **2023**, 473, 145387.

[3] W. Jia, Y. Hou, M. Zheng, M. Zhu, *J. Alloys Compd.* **2017**, 724, 306.

[4] W. Cao, L. Li, K. Chen, X. Huang, F. Li, C. Wang, J. Zheng, X. Hou, Z. Cheng, *Adv. Sci.* **2024**, 2409113.

[5] B. Yang, Y. Liu, W. Li, S. Lan, L. Dou, X. Zhu, Q. Li, C. W. Nan, Y. H. Lin, *Adv. Mater.* **2024**, 2403400.

[6] H. Zhao, W. Cao, D. Han, X. Zhu, C. Liang, C. Wang, C. Wang, *J. Materiomics* **2024**, 10, 947.

[7] C. Wu, Y. Pu, X. Lu, Y. Ning, L. Zhang, B. Wang, Z. Chen, P. Lv, Y. Yang, Z. Wang, *Composites Part B: Engineering* **2024**, 288, 111853.
